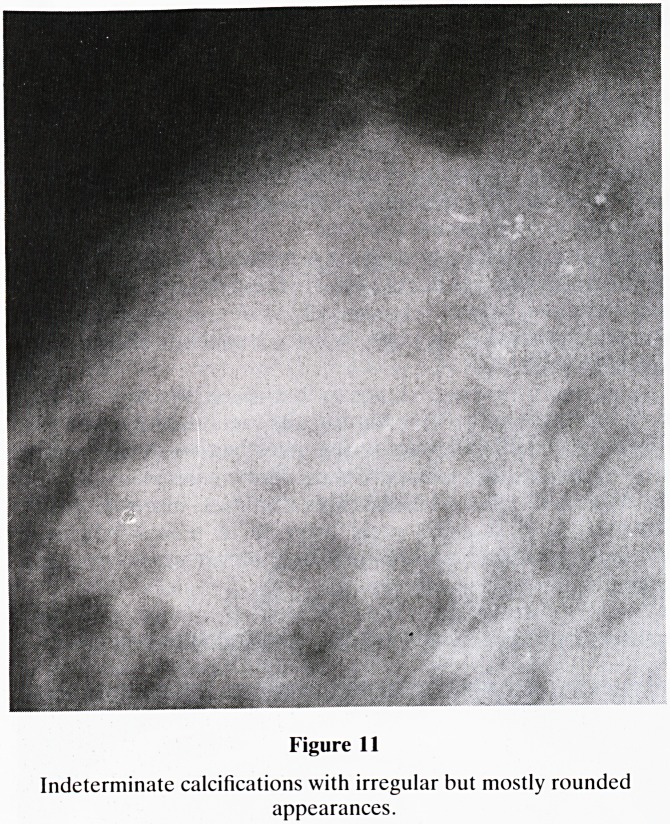# Practical Aspects of Mammography

**Published:** 1989-05

**Authors:** C. R. M. Boggis

**Affiliations:** Consultant Radiologist, Manchester Breast Screening Service, University Hospital of South Manchester


### Practical Aspects of Mammography

**C. R. M. Boggis**
Consultant Radiologist, Manchester Breast Screening Service, University Hospital of South Manchester

### INTRODUCTION

The following guidelines for mammographic evaluation are designed for those without or with limited experience of interpreting mammograms. The aim is to gain confidence with step by step analysis. Knowledge of normal breast anatomy (1, 2) and basic radiological technique is assumed. By using the same method of review each time incorrect analysis can be easily rectified.

### METHOD

Before analysing the information on the mammograms, a few simple, but important rules must be followed.

1. *Display:* Place the mammograms up on the viewer yourself and always in the same manner (figure 1). In this way correct orientation of the routine projections, mediolateral oblique, cranio-caudal and true lateral will be learnt.

   This will also lead to quick, precise identification of each quadrant (figure 2) and allows for easy comparison between both breasts and each quadrant.

2. *Viewing:* The mammograms should be well coned and projected on a variable illuminator. For optimal viewing the use of a magnifying glass and a viewer should be encouraged. The latter cuts down extraneous light and is of particular value in screening. With practice, these tools become indispensable.

3. *Check Radiographic Technique:* Look specifically for:-
   a. Adequate, symmetrical pectoral muscles as far as the nipple.
   b. Inframammary fold.
   c. Radiographic Development—Correct exposure to perceive breast structures i.e. you should just be able to see the skin without a bright light.
   d. Nipple in profile.

4. *Analysis:* Evaluation of information can now begin.

   Firstly determine an impression of overall symmetry from a distance. Look specifically for mass lesions behind the gland disc, this is normally a fatty area. Masses if present behind the nipple, may necessitate surgical removal of the nipple with major cosmetic results. Look for skin thickening and striations extending from the gland tissue to the skin.

**Figure 1**
Format display of mammograms.

**Figure 2**
Identification of the quadrants on mammograms.

**Figure 3a**

Normal glandular appearances.

**Figure 3b**

Fatty glandular appearances to the breast.

**Figure 3c**

Fatty breast.

**Figure 3d**

Prominent duct pattern to the gland tissue.

**Figure 3e**

Nodular appearances to the gland tissue.

**Figure 3f**

Cyst-like appearances to the gland tissue supporting cystic mastopathy; Halo sign arrowed.

**Figure 4a**

Right axillary tail.

**Figure 4b**

Left axillary tail demonstrating asymmetry and the presence of a malignant lesion (arrowed).

5. *Nomenclature of Gland Pattern* (figure 3): A new classification of gland tissue has been formulated (3) and is summarised below.
a. Glandular (figure 3a)—Normal appearances, occurring pre and post menopausally. Be aware of hormone replacement therapy causing gland stimulation after the menopause.
b. Fatty/glandular (figure 3b)—Normal appearances, ongoing growth and involution occurs simultaneously in the breast.
c. Fatty (figure 3c)—Normal involutional changes almost complete.
d. Prominent duct (figure 3d)—Possibly due to duct ectasia or peri-ductal fibrosis and/or inflammation.
e. Nodular (figure 3e)—Due to the prominent duct pattern being very marked and the criss cross pattern of the ducts appearing as nodules in two dimensions.
f. Cyst-like (figure 3f)—Multiple oval opacities, supporting a diagnosis of cystic mastopathy.

**Figure 5**

Large lipoma of fat density (margins arrowed).

Age related changes commonly cause involution of gland tissue with fat replacement. This deposition may be asymmetrical, aggressive, or cause an isolated focus of gland tissue.

7. *Asymmetry:* Compare each quadrant of breast with that of the opposite side. Specifically look for:-
a. Localised increased density—is there an underlying tumour? (figure 4a & b)
b. Architectural disturbance—is there malignant retraction of gland tissue?

Asymmetry of type (b) is strongly suggestive of malignant change, rather than asymmetrical involutionary changes.

8. *Mass Lesions:* When a mass lesion is identified, consider the following factors.

*Density:-* Compare the density of the mass lesion with that of the surrounding gland tissue.
a. greater—solid lesion, possible malignancy
b. equal—benign or malignant
c. less than—fat density in a lipoma (figure 5)
d. complex—if the lesion has malignant features these outweigh the benign properties.

9. *Outlines:* One of the most important discriminants between a benign and malignant lesion is the border of the mass.

**Figure 6**

Large central mass with short spiculations and malignant type calcifications.

**Figure 7a**

Mediolateral oblique projection with long spiculations in the upper half with no solidity to the central mass (arrowed).

**Figure 7b**

Cranio-caudal projection shows no spiculations present.

a. Clear, well defined, halo sign (see figure 3f)—most likely benign disease
b. Blurred, irregular comet tail—suspicious of malignancy
c. Spiculations, particularly short—commonly malignant

Benign mass lesions are usually well-defined, round, oval or occasionally lobulated. They may have a halo sign, which is a fine surrounding lucent line of fat density. The most likely benign lesions are, fibroadenomata in younger women and cystic lesions in perimenopausal women. However, the well defined benign type lesion in a post menopausal fatty breast should be interpreted with care as in this scenario, there are malignant lesions that mimic benign masses, such as mucoid and medullary carcinomas.

10. Spiculations: Analysis of spiculations should be made with respect to their length and relationship to a central mass.
a. Short spiculations that are associated with a solid central mass is highly indicative of malignant change (figure 6). Look for supporting evidence and secondary signs of malignancy (distortion, skin thickening).
b. Long spiculations if malignant should cause significant architectural disturbance. Long, non retractile spiculations, together with a mixed density centre, point towards a benign cause e.g. radial scar, sclerosing adenosis, (figure 7a & 7b). Thickening of Cooper's lignaments and fibrous strands to the skin can be caused by surgical scarring lending to difficulty in diagnosis.

11. *Size:* Measure the size of the lesion, both the central core and length of spiculations. This information aids surgical planning as wide local excision or simple mastectomy may depend on the size of the lesion which can differ significantly clinically and radiologically. Radiologically it can not be determined whether the spiculations are due to tumour infiltration or a scirrhous reaction.

12. *Location:* Place the lesion in a quadrant (figure 1) and measure its distance from the nipple. With this format of reporting it is easier to convey the correct information to colleagues. The analogy of the clock face may lead to confusion.

Remember, that the lymphatic drainage of the inner halves of the breasts are to the internal mammary chain, thus, these nodes are both impalpable clinically and unavailable for examination at surgery. This means that inner half lesions may have a relatively poorer prognosis than outer half lesions.

13. *Alignment:* Try to determine whether an identified lesion is anatomically segmental and due to a typically segmental disease such as cystic hyperplasia.

14. *Multiplicity:* Benign diseases are frequently multiple e.g. cysts, fibroadenomas. BUT, more than one carcinoma may occur and this warrants bilateral mammography and the routine search for a second or adjacent lesion.

**Figure 8**

Large dense calcifications (duct pearls).

**Figure 9**

Fine, irregular, mixed malignant type calcifications in the left lower half.

15. *Calcifications:* Do not focus on calcifications at the expense of other valuable and diagnostic mammographic features that may be present. The analysis of calcifications fall into four groups:-
a. Benign type (figure 8)—large, chunky and very dense; e.g. duct pearls, (smooth and round) and those associated with fibroadenoma or fat necrosis, (irregular).
b. Malignant type (figure 9)—very fine, small irregular in shape, size and density and termed microcalcifications.
c. Lobular type (figure 10)—these are groups of small, round calcifications on the cranio-caudal, view. becoming typically crescentic on the oblique projection.
d. Indeterminate type (figure 11)—clustered, mixed irregular calcifications. This type requires surgical biopsy and histological analysis.

16. *Distribution:-*
a. Small clusters of calcifications tend towards malignancy.
b. Segmental distribution may represent malignant change such as lobular carcinoma. However, lobular type is also seen in cystic hyperplasia.

17. *Skin thickening and tethering:* Skin thickening is seen with malignancy, surgical treatment, radiation therapy, previous infection and oedema. If due to the latter look for increased numbers and thickness of the Cooper's liagments.

18. *Axillary Lymph Nodes:* Lymph nodes are radiologically well defined often lobulated with a relatively lucent centre. They are found within the breast initially in approximately 25% of women and usually easily recognised.

19. *Interpretation:* Analyse the findings and this should lead to a diagnosis of normality, benign or malignant disease.

**Figure 10**

The diagramatic representation of the calcifications in the mediolateral oblique and cranio-caudal view.

Use of a form (table 1) may be helpful when beginning to read mammograms. Correlation between radiological and histopathological findings is important as a learning exercise and in obtaining feedback of radiological opinions.

Communication between all the specialities involved in the diagnosis and management of breast disease is to be encouraged. This leads to greater understanding of disease processes by different specialty groups.

Mammographic interpretation necessitates thorough systematic appraisal and requires excellent radiographic technique.

**Figure 11**

Indeterminate calcifications with irregular but mostly rounded appearances.

**Table 1**

**Mammography Analysis Scheme**

| *Name* | *Number* |
|---|---|
| Radiographic technique | |
| Review Areas | |
| Gland Pattern | |
| Asymmetry | |
| *Mass/Shape* | |
| Density | |
| Border | |
| Size | |
| Location | |
| Multiplicity | |
| Calcifications | |
| Other | |
| Inference | |
| *Calcifications* | |
| Form | |
| Distribution | |
| Inference | |
| *Mammographic diagnosis* | |

### ACKNOWLEDGEMENTS

I would like to thank my Teachers in Mammography, Dr Geoffrey Hartley, Dr David Asbury of University Hospital South Manchester. I would also like to thank Mrs Susan Oatway and Mrs Ann Walter for their typing and patience during the preparation of this paper and the Medical Illustrations Department at the University Hospital of South Manchester.

### REFERENCES

1. 'Teaching Atlas of Mammography'. L. Tabar and P. Dean, George Thieme Verlag. 1985.
2. 'An Atlas of the Breast' Jean-Louis Lamarque Wolfe Medical Publications 1984.
3. Radiological Nomenclature in Benign Breast Change. (Proposals by the Breast Group of the Royal College of Radiologists).

1989 Clinical Radiology (in press).

## Figures and Tables

**Figure 1 f1:**
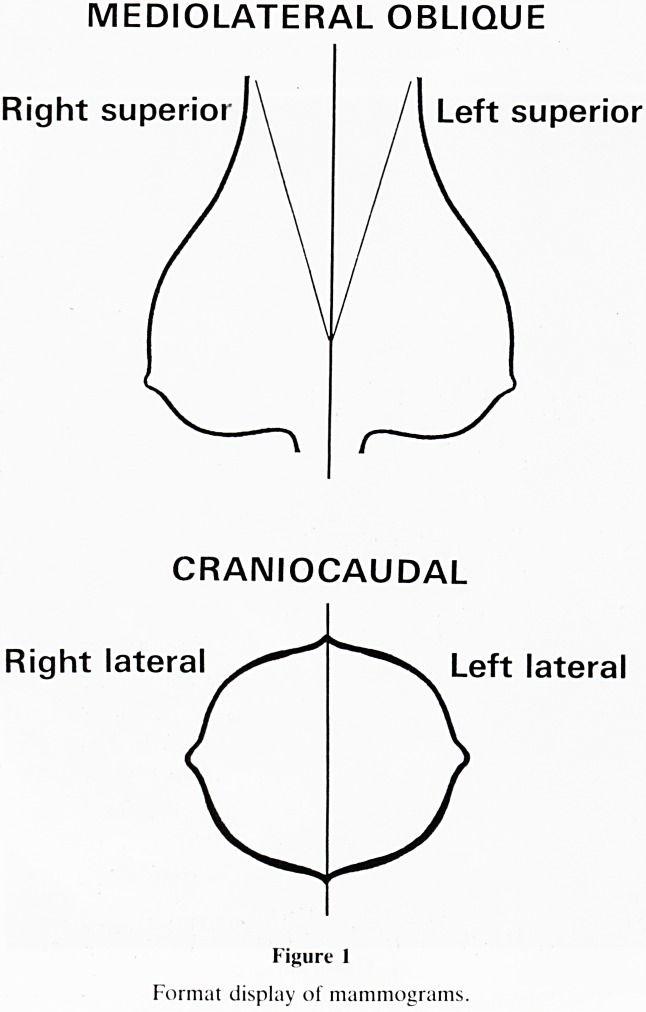


**Figure 2 f2:**
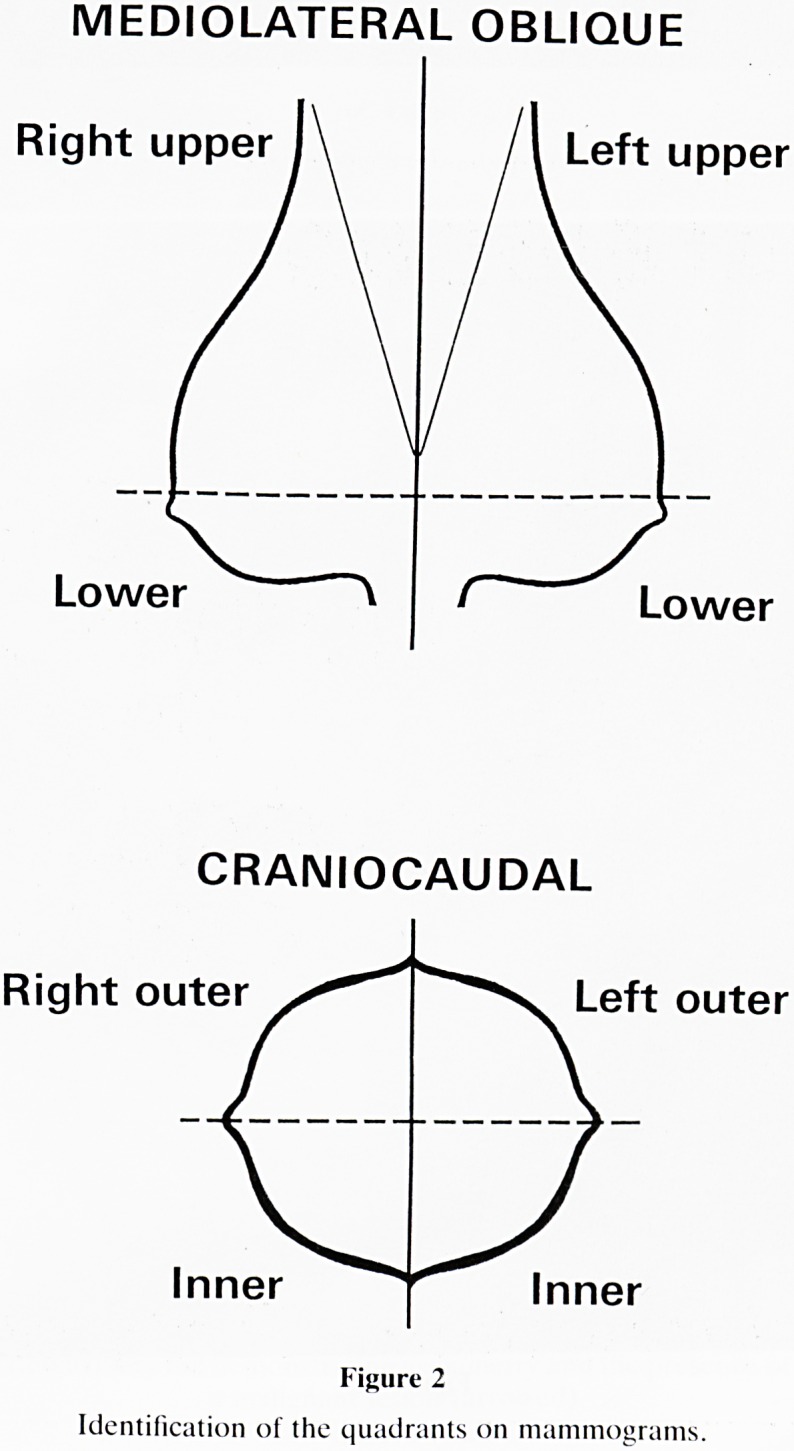


**Figure 3a f3:**
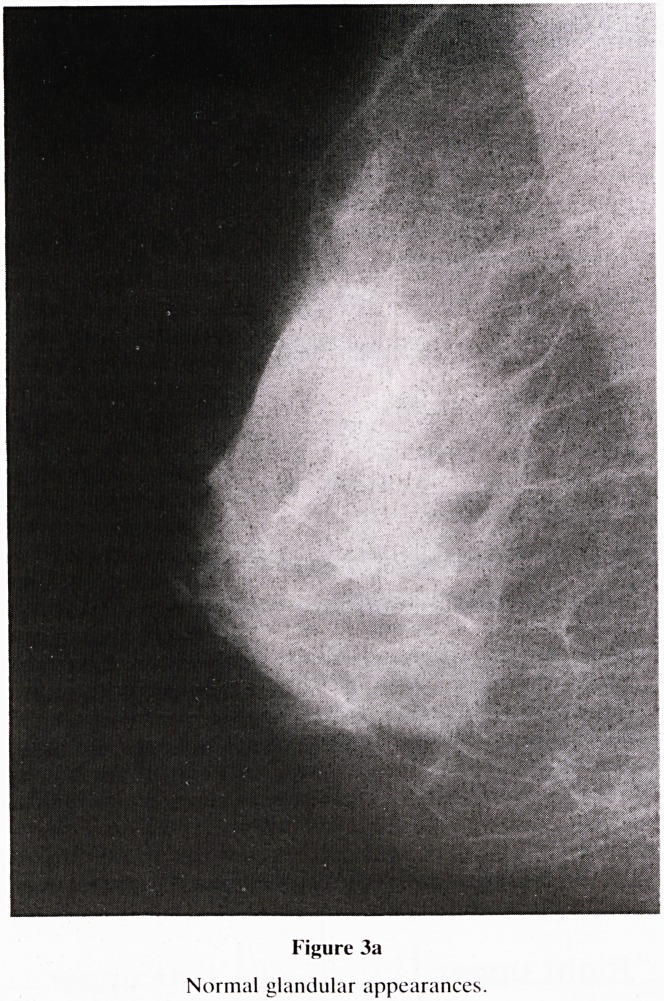


**Figure 3b f4:**
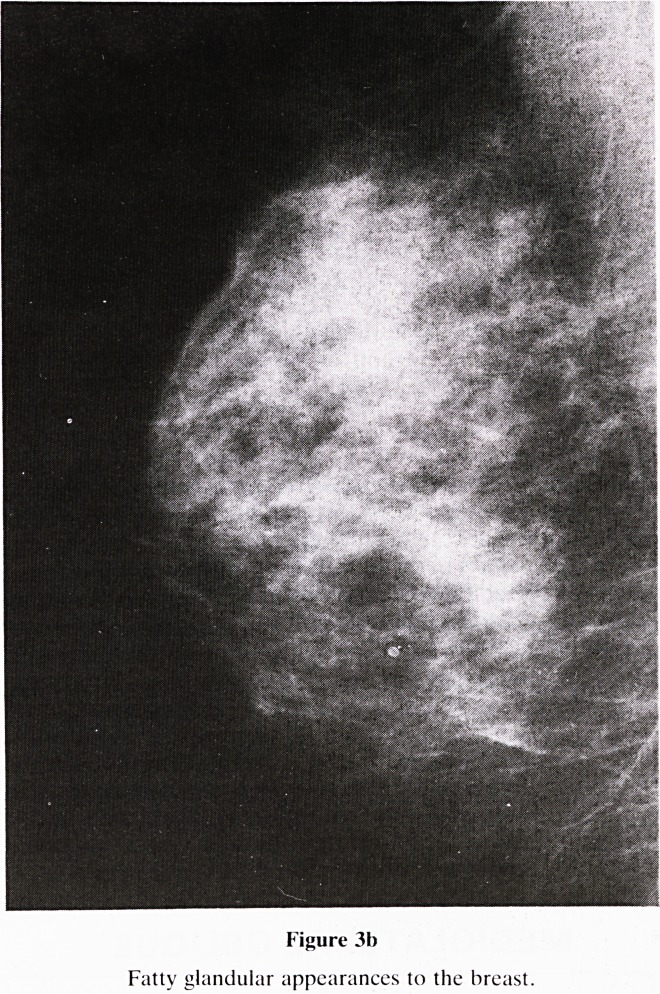


**Figure 3c f5:**
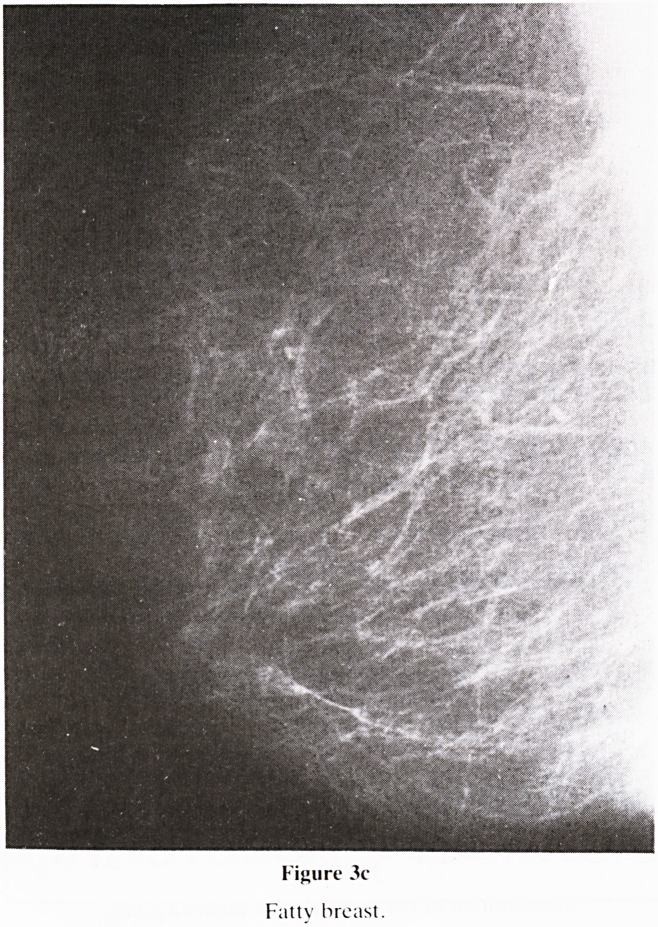


**Figure 3d f6:**
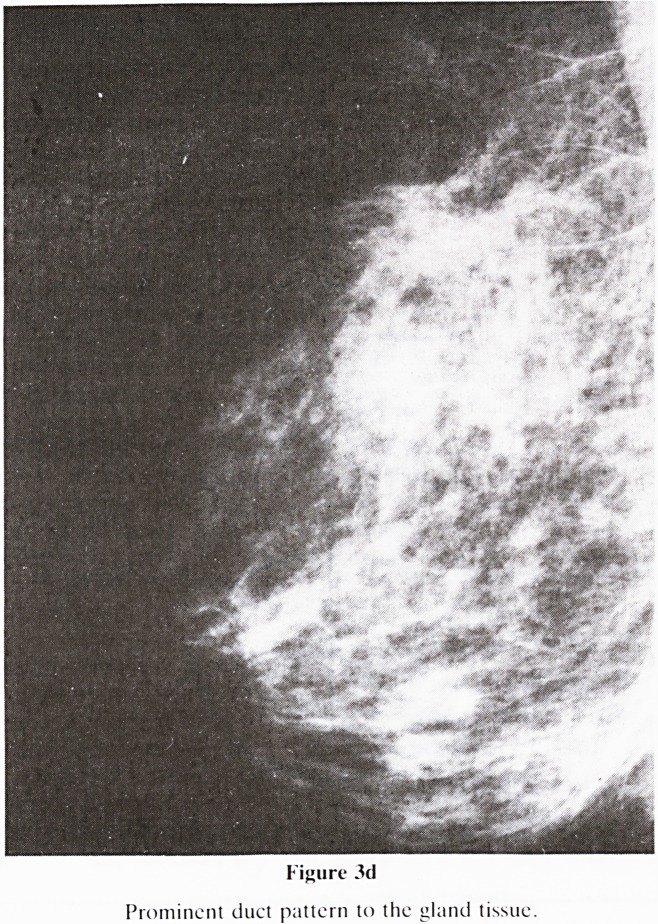


**Figure 3e f7:**
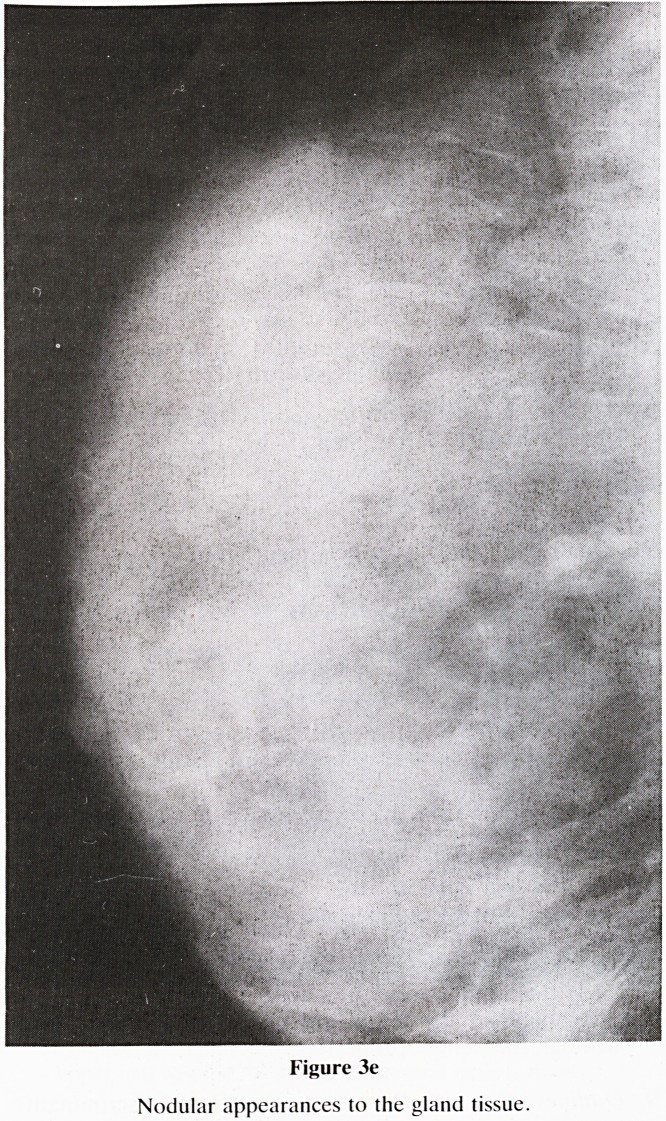


**Figure 3f f8:**
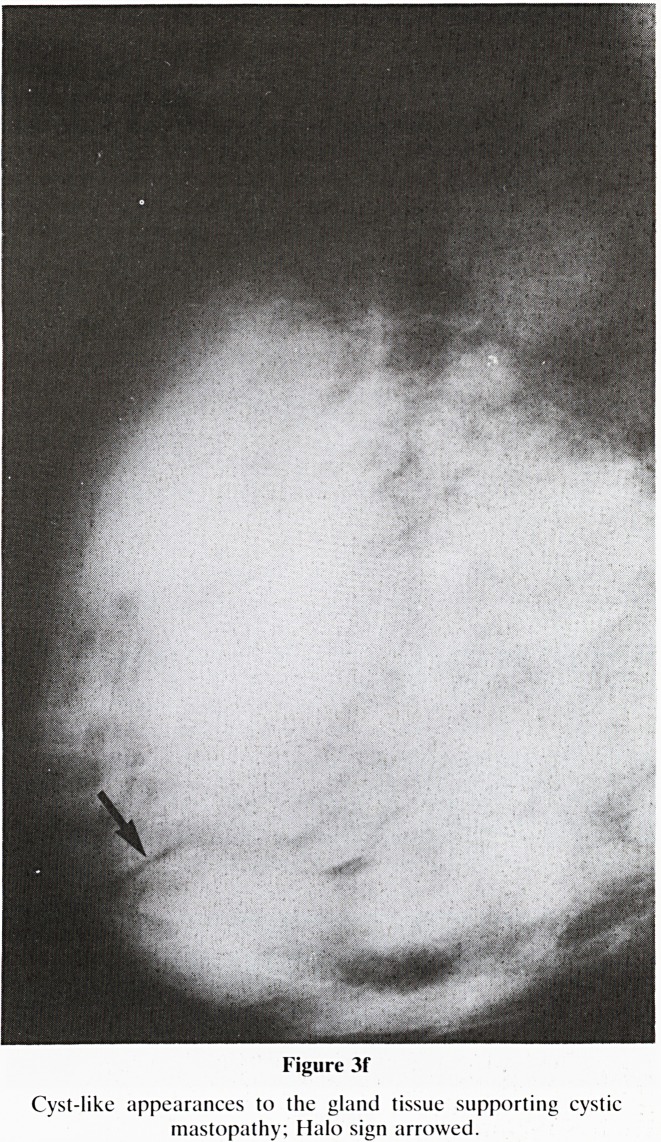


**Figure 4a f9:**
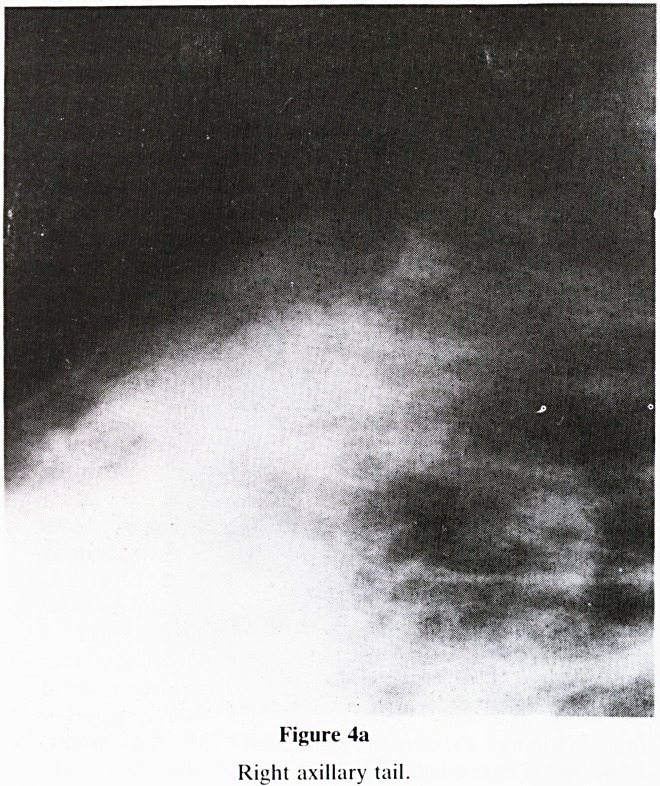


**Figure 4b f10:**
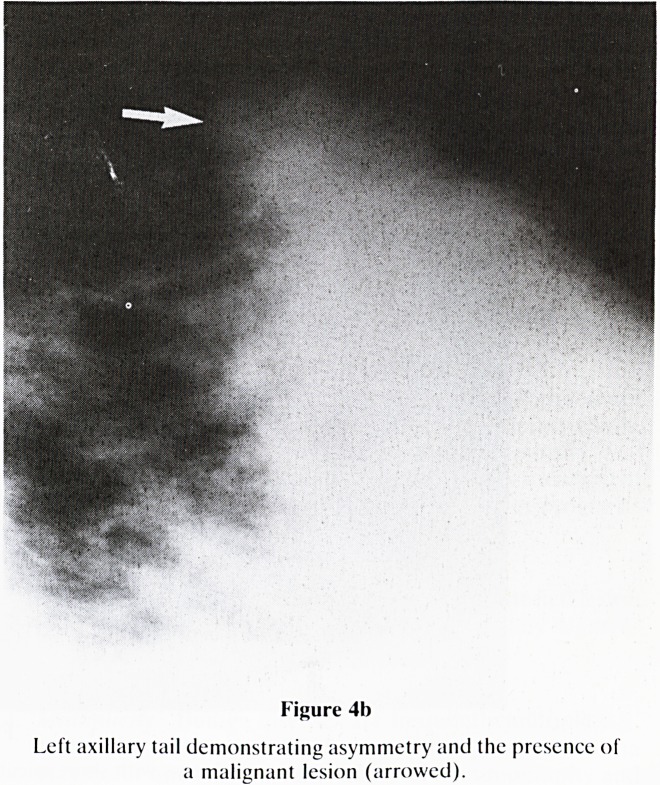


**Figure 5 f11:**
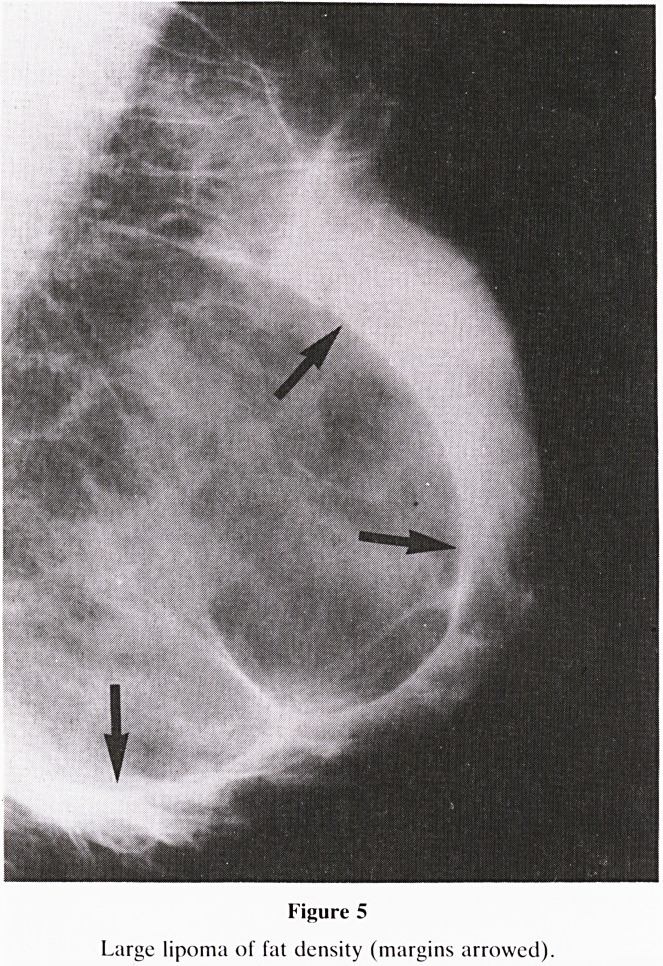


**Figure 6 f12:**
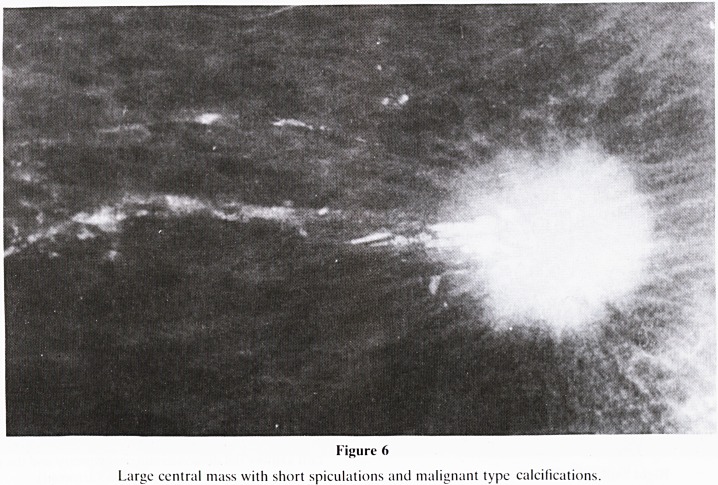


**Figure 7a f13:**
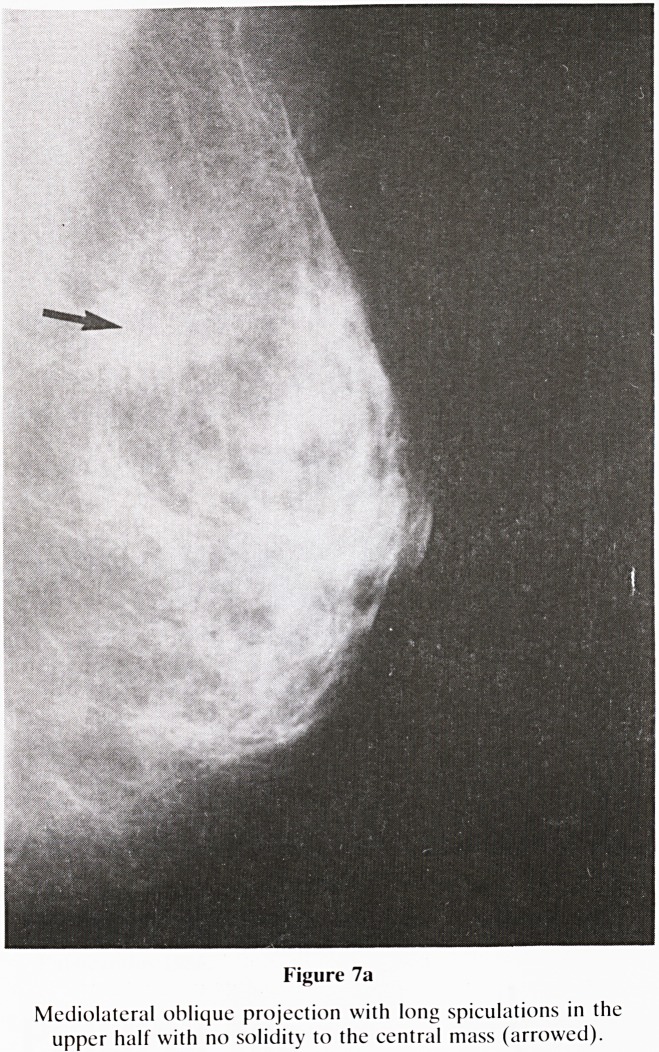


**Figure 7b f14:**
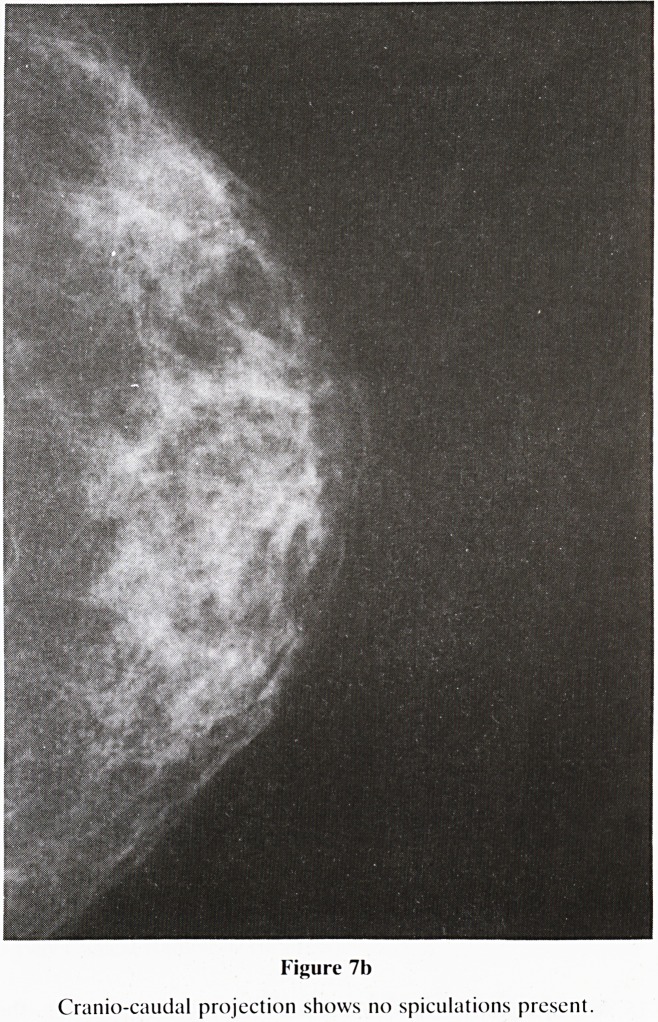


**Figure 8 f15:**
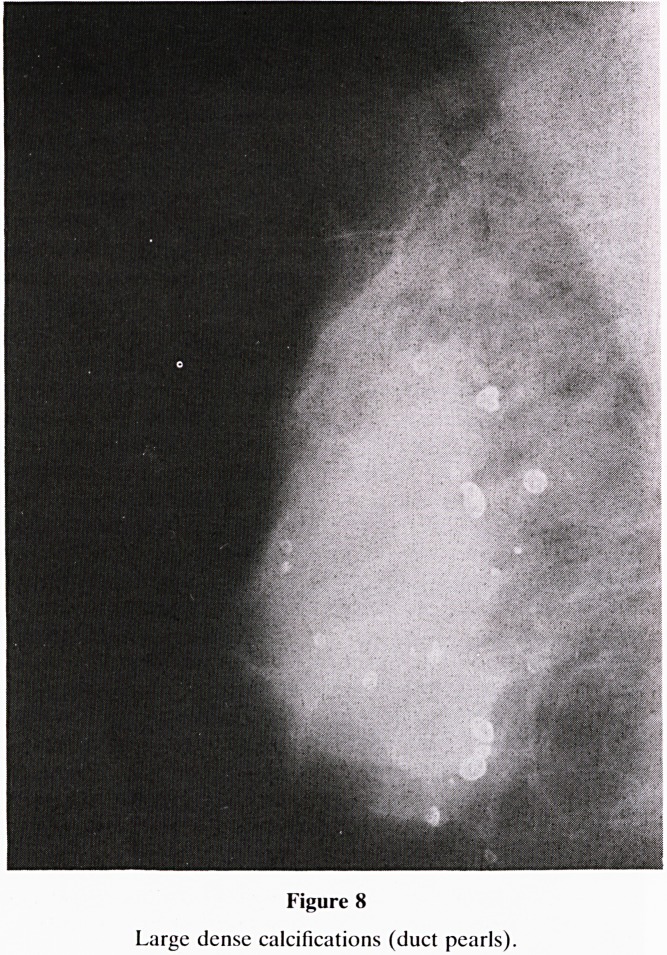


**Figure 9 f16:**
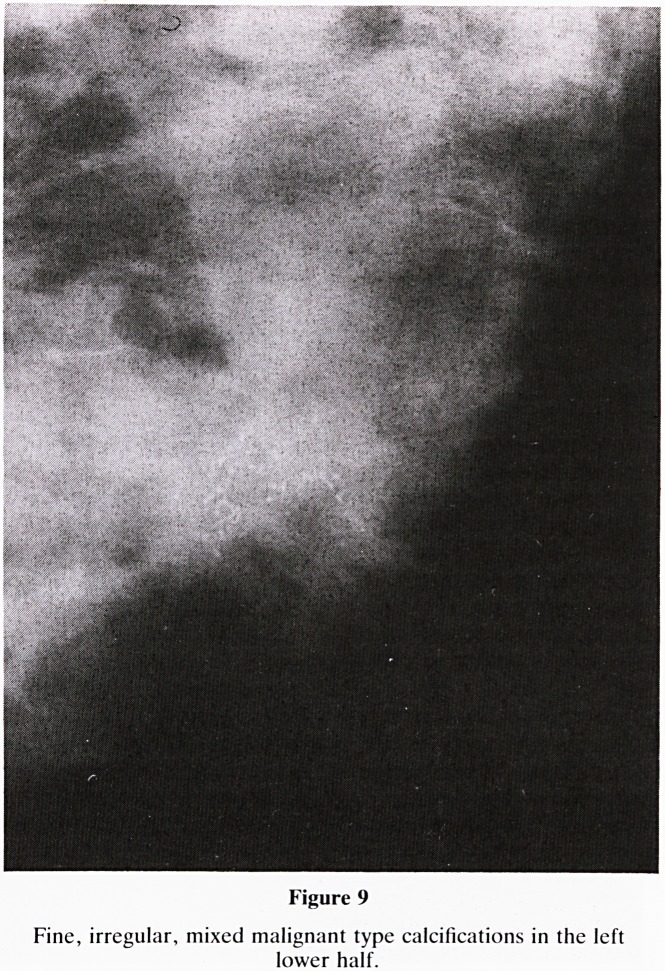


**Figure 10 f17:**
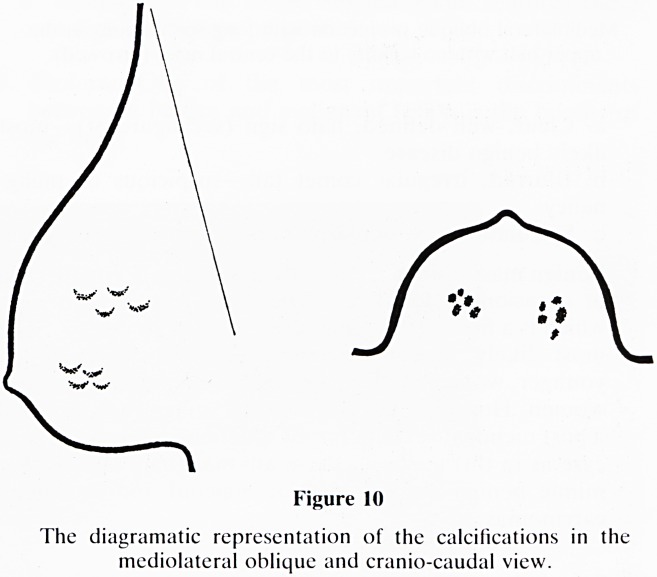


**Figure 11 f18:**